# Redefining pancreatogenic diabetes mellitus through molecular, imaging, and AI-driven evidence

**DOI:** 10.3389/fendo.2025.1749805

**Published:** 2026-01-23

**Authors:** Imran Rashid Rangraze, Mohamed El-Tanani, Adil Farooq Wali, Rasha Babiker, Syed Arman Rabbani, Ismail I Matalka, Shakta Mani Satyam, Ashot Avagimyan, Karolina Hoffmann, Ioannis Ilias, Sorina Ispas, Maggio Viviana, Anna Paczkowska, Manfredi Rizzo

**Affiliations:** 1 RAS AL KHAIMAH (RAK) College of Medical Sciences, RAK Medical and Health Sciences University, Ras Al Khaimah, United Arab Emirates; 2 RAS AL KHAIMAH (RAK) College of Pharmacy, RAK Medical and Health Sciences University, Ras Al Khaimah, United Arab Emirates; 3 RAS AL KHAIMAH (RAK) Medical and Health Sciences University, Ras Al Khaimah, United Arab Emirates; 4 Yerevan State Medical University after M. Heratsi, Yerevan, Armenia; 5 Isfahan Cardiovascular Research Centre, Cardiovascular Research Institute, Isfahan University of Medical Sciences, Isfahan, Iran; 6 Department and Clinic of Internal Diseases and Metabolic Disorders, Poznan University of Medical Sciences, Poznań, Poland; 7 Department of Endocrinology, Diabetes and Metabolism, Elena Venizelou Hospital, Athens, Greece; 8 Department of Anatomy, Faculty of General Medicine, “Ovidius” University, Constanta, Romania; 9 School of Medicine, PROMISE Department of Health Promotion Sciences Maternal and Infantile Care, Internal Medicine and Medicinal Specialties, University of Palermo, Palermo, Italy; 10 Department of Pharmacoeconomics and Social Pharmacy, Poznan University of Medical Sciences, Poznań, Poland

**Keywords:** AI diagnostics, biomarkers, exocrine pancreatic insufficiency, pancreatogenic diabetes, precision medicine, reclassification, pancreatogenic diabetes mellitus

## Abstract

**Background:**

Pancreatogenic Diabetes Mellitus (PDM), denoting diabetes secondary to fibro-inflammatory and structural pancreatic injury, represents a distinct yet under-recognised clinical entity. Current classification systems frequently subsume this condition under ―other specific types‖ of diabetes, contributing to misdiagnosis, therapeutic misdirection, and suboptimal outcomes.

**Objectives:**

This review critically analyses contemporary evidence regarding the pathogenesis, diagnostic framework, metabolic consequences, and therapeutic requirements of pancreatogenic diabetes. We propose a mechanism-based, precision-oriented clinical model integrating structural, functional, and emerging biomarker evidence.

**Methods:**

An integrative review of recent literature was conducted, focusing on pancreatic pathophysiology, endocrine–exocrine interplay, diagnostic imaging modalities, molecular biomarkers, radiomics, and artificial intelligence–assisted analytics.

**Key findings:**

Pancreatogenic Diabetes Mellitus is characterised by progressive insulin deficiency, impaired glucagon counterregulation, and exocrine pancreatic insufficiency, often accompanied by malnutrition and glycaemic instability. A structured diagnostic framework integrating pancreatic imaging, endocrine–exocrine functional testing, and autoimmune exclusion improves classification accuracy. Management requires coordinated insulin therapy, pancreatic enzyme replacement, nutritional optimisation, and surveillance for skeletal and malignant complications. Emerging radiomic and AIdriven tools may enhance early detection and risk stratification, although prospective validation remains necessary.

**Conclusion:**

Pancreatogenic Diabetes Mellitus warrants clearer mechanistic recognition within global diabetes classification frameworks. Harmonised diagnostic criteria, translational research integration, and multidisciplinary care pathways are essential to improve patient outcomes and advance precision medicine in this domain.

## 1 Introduction

Diabetes mellitus due to disorders of the endocrine and exocrine pancreas, such as chronic pancreatitis, pancreatic neoplasms, pancreatic surgery, and cystic fibrosis, has historically been known as Type 3c Diabetes Mellitus (T3cDM) (1, 2). Evidence suggests that T3cDM comprises mostly 5%-10% of the overall population with diabetes, but frequently remains silent and has been dismissed as Type 2 Diabetes Mellitus (T2DM) (3, 4).

To contextualise why this condition has received limited attention within diabetes literature, it may be useful to briefly consider the historical development of the classification of diabetes.

### 1.1 Historical context: the evolution of diabetes classification

Over the course of the last four decades, the classification of diabetes has seen a great deal of innovation. In 1980 the World Health Organization (WHO) distinguished diabetes as being either insulin-dependent, or non–insulin-dependent (3–5). By the late 1990s, with advances in diabetes care, the WHO and American Diabetes Association (ADA) (6, 7) endorsed a four-part, mechanistic classification system with diabetes being subdivided into Type 1, Type 2, and gestational diabetes, with a large catch-all category of “other specific types.” Although this system of classification served to better identify monogenic and secondary causes of hyperglycaemia, it continued to classify pancreatogenic diabetes along with a number of disparate conditions, failing to recognize its unique amalgam of endocrine deficiency, exocrine failure, and metabolic derailment.

**Figure 1:**
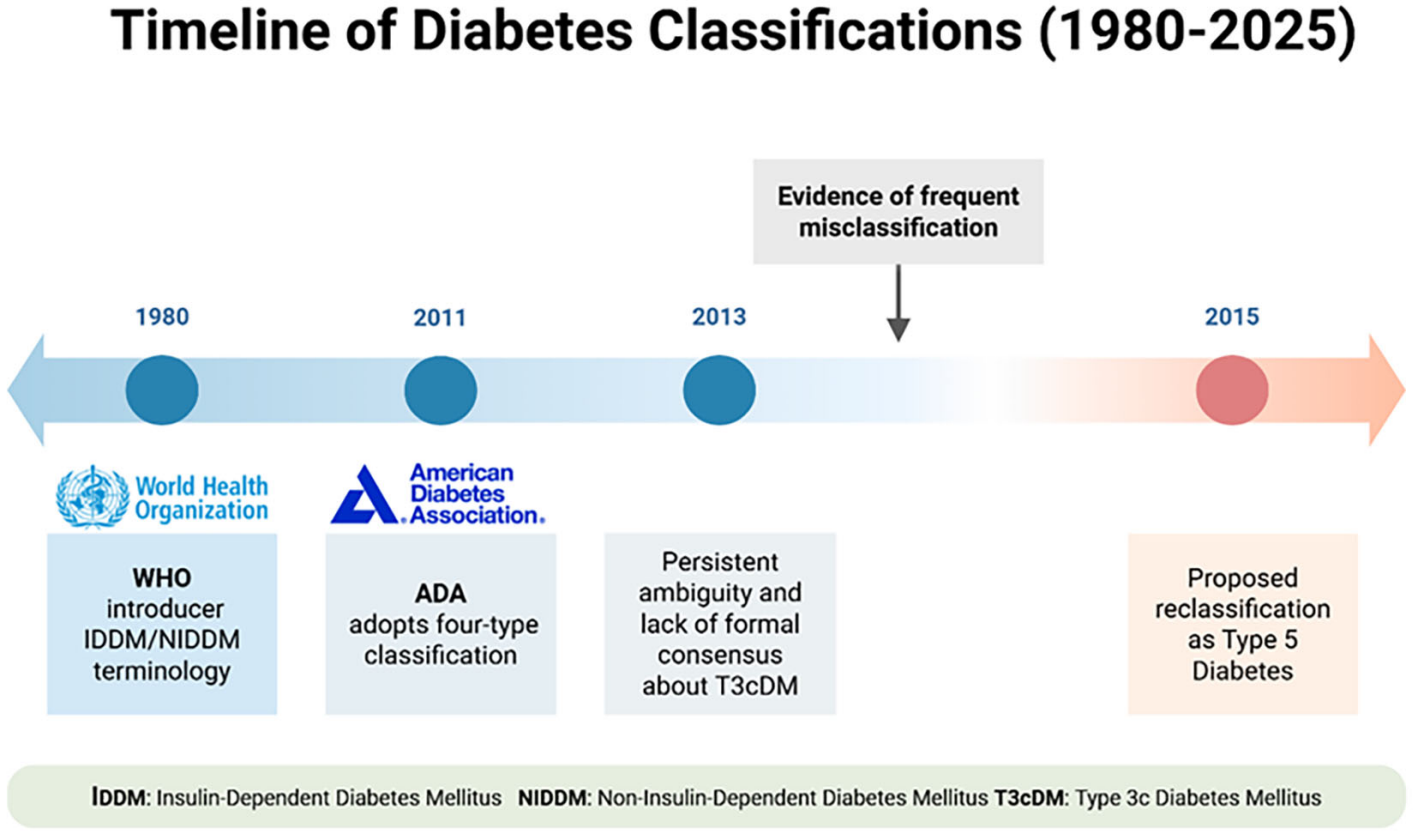
Timeline of diabetes classifications (1980–2025). The figure summarizes key milestones: WHO’s adoption of IDDM/NIDDM terminology (1980), ADA’s four-type classification (2011), recognition of frequent misclassification and ambiguity about Pancreatogenic diabetes (formerly T3cDM) (2013), and the proposal to reclassify it as Pancreatogenic diabetes (2015). *Created in BioRender. Babiker, R. (2025)* *https://BioRender.com/z4inyhx*.

Ewald and Bretzel (3) and Hart et al. (8) have proposed that the condition be formally recognised as Type 3c diabetes mellitus (3, 8) due to its distinct physiology and clinical course. However, despite increasing academic support, PDM remains excluded from international diagnostic and coding standards (**Figure 1**).

### 1.2 Current gaps in recognition and clinical practice

There are serious clinical implications for the continued usage of antiquated nomenclature. Up to 78% of patients with diabetes after pancreatitis are inappropriately treated with management strategies stressing insulin sensitization and avoidance of insulin replacement and nutritional rehabilitation (9). PDM differs from T2DM by having an absolute insulin deficiency, exocrine pancreatic insufficiency and malnutrition, which requires insulin therapy, pancreatic enzyme replacement, micronutrient supplementation, and cancer surveillance (3, 5, 10).

The missing specific diagnostic codes in electronic health records (EHRs) grossly underestimates the prevalence of the disease, the inaccuracy of public health data, and limits the range of clinical knowledge. Likewise, medical education and continuing medical education (CME) programs rarely cover the integration of endocrine and exocrine functions, thus sustaining diagnostic inertia in clinical practice.

### 1.3 Clinical impact and epidemiologic urgency

Epidemiological studies indicate that PDM represents a significant portion of all diabetes cases (11). Owing to a two- to three-fold increased risk of substantial hypoglycemia, deficiencies in fat-soluble vitamins, osteoporosis, and cancer-related death, PDM patients living with T2DM suffer adversities (12).

Epidemiological estimates (i.e. prevalence, misclassification rates, mortality estimates) for pancreatogenic diabetes streams primarily from disparate study groups and thus should be examined with specific clinical reasoning. Approximately 5-10% estimates from chronic pancreatitis cohorts and post-pancreatectomy series, and over 70-80% high misclassification rates have been reported from European registry-based and tertiary care studies. In addition, increased mortality risk is from selected wealthy high-income settings disease specific from disease specific populations and not from a more generalized global cohort. These differences highlight the risks of overgeneralization and the considerable gaps in epidemiological data from low and middle income countries. In these countries, the burden of disease is more likely to be underestimated (**Table 1**).

**Table 1:** Key epidemiological studies informing prevalence, misclassification, and outcomes in pancreatogenic diabetes.

Outcome reported	Approximate estimate	Study population	Study design	Geographic context	Outcome reported
Prevalence	5–10%	Chronic pancreatitis (CP), post-pancreatectomy	Cohort studies	Europe, North America	Prevalence
Misclassification	~70–80%	Registry-based diabetes populations	Retrospective registries	Europe	Misclassification
Mortality	2.5–3-fold increase	CP-associated diabetes	Longitudinal cohorts	High-income settings	Mortality

### 1.4 Objective of this review

This review collates the recent literature on the evolving evidence supporting the discourse on the recognition of PDM as a new clinical condition. It describes the etiological diversity, fundamental pathways and diagnostic features that make up pancreatogenic DM, and presents a complete clinical proposition that involves the amalgamation of molecular, imaging and AI-based diagnostics with customised medicine. It aims to inform clinicians and decision-makers about the need to officially include PDM as one of the subclasses in the international classification of diabetes.

In proposing PDM, we do not intend a simple semantic renaming of Type 3c diabetes mellitus, nor do we suggest that all historical descriptions of pancreatogenic diabetes are inadequate. Rather, Pancreatogenic Diabetes Mellitus is advanced as a mechanistically expanded and clinically operational redefinition of pancreatogenic diabetes. While T3cDM has traditionally served as a descriptive category encompassing diabetes secondary to pancreatic disease, it has remained limited by heterogeneous definitions, inconsistent diagnostic criteria, and lack of integration of modern pathophysiological, imaging, and biomarker-based insights. The PDM framework builds upon the foundational concept of pancreatogenic diabetes while incorporating contemporary evidence related to fibro-inflammatory pancreatic injury, endocrine–exocrine cross-talk, dynamic β-cell failure, and emerging diagnostic tools, thereby offering a precision-oriented model for classification and management.

### 1.5 Controversy of nomenclature and rationale for the term “pancreatogenic diabetes mellitus”

The proposal to formally adopt the term Pancreatogenic Diabetes instead of the historically used Type 3c Diabetes Mellitus warrants careful discussion, as numerical classification in diabetes carries both scientific and health-policy implications (**Table 2**). We acknowledge that introducing a new numerical subtype may raise concerns regarding nomenclature continuity, potential confusion, and hierarchical equivalence with Type 1 and Type 2 diabetes.

**Table 2:** Comparison between traditional Type 3c diabetes mellitus and the proposed Pancreatogenic diabetes framework.

Domain	T3cDM (Traditional)	Pancreatogenic diabetes (Proposed Framework)
Conceptual basis	Descriptive, etiology-based	Mechanism-driven, precision-oriented
Scope	Diabetes secondary to pancreatic disease	Expanded pancreatogenic diabetes spectrum
Pathophysiology	Heterogeneous, poorly standardized	Fibro-inflammatory paradigm; Triple-Hit model
Endocrine dysfunction	Insulin deficiency emphasized	Dynamic insulin ± glucagon dysfunction
Exocrine integration	Often underemphasized	Central to diagnosis and management
Diagnostic approach	Exclusion-based, inconsistent	Tiered framework (imaging, function, biomarkers)
Role of imaging	Supportive	Central (MRCP/EUS; AI as future adjunct)
Biomarkers	Limited use	Integrated (C-peptide continuum, nutrition, inflammation)
Clinical utility	Retrospective label	Forward-looking care pathway
Alignment with precision medicine	Limited	Explicitly aligned

As far as diabetes sub classification terminology is concerned, from a purely theoretical and abstract perspective, it is academically acknowledged that the sub classifications of diabetes is theoretically, numerically, and mathematically incoherent. For many, the term “Type 3 diabetes” has become a popular, albeit informal, and quasi unacademic description of dementia and Alzheimer’s disease as a state of cerebral insulin resistance, a notion, albeit distinctly unrecognized, by the American Diabetes Association (ADA) and the World Health Organization (WHO). It is also worth noting that, “Type 4 diabetes” has been used soeky and informally in the older academic and regional texts as a more informal, quasi unacademic description of diabetes associated with pregnancy, malnutrition or undernutrition, and has been a matter of no formal academic consensus. Attempts to redefine these terms in the endocrinology field and diabetes sub terminology, may potentially create more ambiguity. In this regard, Pancreatogenic diabetes is far more relevant than other terms, and this could potentially create more openness in a diabetes nomenclature. The term is not based on indicating the absence of other terms of greater relevance.

Another concern focuses on the assumption that pancreatogenic diabetes can only be considered a complication of pancreatic injury and, as a result, should continue being categorized as an “other specific type” of diabetes. Although the “primary” injury in PDM is technically a disturbance of the pancreas, be it in the form of chronic pancreatitis, pancreatic cancer, cystic fibrosis, or surgery, being secondary in origin does not exclude the classification as “primary” along with a distinct, reproducible identity that results in a metabolic disorder of the respective clinical complication of interest. In this case, PDM is marked by an abnormal and rare combination of clinical features, which include the absence of insulin, impaired glucagon counter-regulation, exocrine pancreatic insufficiency, malnutrition, dysglycemia, and an increased risk of cancer. The range of features of this disorder differentiates it from the other autoimmune forms (Type 1 diabetes) and the insulin-resistant forms (Type 2 diabetes), necessitating a unique approach toward diagnosis and management.

Importantly, the continued placement of pancreatogenic diabetes within the ADA category of “other specific types” has contributed to systematic under-recognition and misclassification, most commonly as advanced Type 2 diabetes. In contemporary clinical practice, patients with diabetes following pancreatic disease are frequently coded under non-specific ICD-10 categories such as E13 (“other specified diabetes mellitus”) or E11 (“Type 2 diabetes mellitus”), resulting in omission of pancreatic enzyme replacement therapy, inadequate nutritional rehabilitation, limited access to continuous glucose monitoring, and insufficient surveillance for pancreatic malignancy.

From a health-policy perspective, the proposal of Pancreatogenic diabetes should be understood as a strategic classification construct rather than a purely semantic exercise. A distinct category has the potential to improve disease visibility within electronic health records, enable accurate epidemiological tracking, facilitate reimbursement for multidisciplinary care (including enzyme replacement and nutrition therapy), and support the development of registries and clinical pathways tailored to this high-risk population. Similar shifts in disease recognition—such as the formal differentiation of heart failure with preserved ejection fraction—have demonstrated that reclassification can materially influence clinical outcomes, research prioritization, and resource allocation.

In this context, we propose Pancreatogenic diabetes as a mechanism-based and care-oriented classification that addresses the distinct pathophysiology and clinical needs of pancreatogenic diabetes, while also being fully compatible with the ADA and WHO. Rather, we seek to expound the recognition of diabetes distinct from Type 1 and Type 2 diabetes, as this poses the greatest and most policy-relevant challenge in the optimal recognition, diagnosis, and management of this particular diabetes.

## 2 Etiology and pathogenesis

PDM arises due to damage sustained to the exocrine pancreas alongside chronic inflammation, leading to continued injury of the integrated endocrine–exocrine axis (13).

### 2.1 Primary etiological conditions

Numerous pancreatic conditions that share similar pathophysiological changes result in PDM.

- Chronic pancreatitis (CP) is responsible for roughly 80% of PDM. There is continuous stimulation of pancreatic stellate cells which induces fibrosis, duct obstruction, and acinar atrophy. Ischemia and the injurious cytokines TGF-β, IL-1β, TNF-α involved in the inflammatory response cause the further loss of both β- and α-cells. Clinical symptoms such as weight loss and steatorrhea often come before the hyperglycemia starts (14).
- Pancreatic ductal adenocarcinoma (PDAC) is linked to diabetes that can develop before or after cancer. Tumours release adrenomedullin and IL-6 which can cause hepatic insulin resistance and β-cell dysfuction resulting in paraneoplastic diabetic syndrome. The development of diabetes after 50 years of age is reason enough to thoroughly investigate the pancreas (15).
- Cystic Fibrosis (CF) is the result of mutations in the CFTR gene that cause the secretions to be thick, leading to ductal obstruction. This results in loss of pancreatic islet function that causes exocrine pancreatic insufficiency. CF diabetes is atypical because it has features of both type 1 and type 2 diabetes; however, it is distinctly characterised by severe malnutrition and an unusual preservation of glucagon secretion (16).
- Conditions subsequent to pancreatectomy surgery involve surgical excision of both beta and alpha cells and the resultant disruption of the islet microcirculation. There is a compounding relationship between the level of hyperglycemia and the amount of pancreatic tissue lost. Total pancreatectomy results in brittle, insulin-dependent diabetes mellitus (17).
- Additional Causes: Rare explanations include autoimmune pancreatitis, hemochromatosis, pancreatic agenesis or lipomatosis, and certain genes (SPINK1, PRSS1, CTRC) that increase the risk of chronic inflammation (18).

**Table 3** presents a comparative analysis of the etiology, pathophysiology, and clinical characteristics of the principal diabetes subtypes.

**Table 3:** Comparative etiology, pathophysiology and clinical features of major diabetes subtypes.

Feature	Type 1 DM (T1DM)	Type 2 DM (T2DM)	Pancreatogenic DM	MODY
Etiology	Autoimmune β-cell destruction, triggered by environment in genetically predisposed	Genetic + lifestyle; obesity, inactivity, age	Secondary to pancreatic exocrine diseases (chronic pancreatitis, surgery, CF)	Single-gene defects affecting β-cells; autosomal dominant
Pathophysiology	Absolute insulin deficiency due to immune-mediated β-cell loss	Insulin resistance + progressive β-cell dysfunction	Loss of insulin, glucagon, enzymes due to structural damage	Isolated β-cell dysfunction; impaired glucose sensing or secretion
Genetics	Polygenic; strong HLA-DR3, DR4-DQ8 links; INS, PTPN22	Polygenic; >100 loci (e.g. TCF7L2), strong family history	No direct genetics; linked to underlying disease (e.g. CFTR in CF)	Monogenic; HNF1A (MODY3), GCK (MODY2), HNF4A, HNF1B
Typical Age at Onset	<30 years, often childhood/adolescence	>40 years, but younger with obesity	30–60 years, with pancreatic disease onset	<25 years; often incidental discovery
Body Habitus at Onset	Lean or normal weight; recent weight loss	Overweight or obese with central adiposity	Often lean or underweight from malabsorption	Normal BMI
Autoimmunity	Islet autoantibodies present (GAD, IA-2, ZnT8, IAA)	No autoimmunity	No autoimmunity	No autoimmunity
Insulin Resistance	Minimal at onset, may develop later	Marked insulin resistance in muscle, liver	CP: Variable (inflammatory-mediated) PDAC: High (paraneoplastic, hepatic) CF: Variable Post-pancreatectomy: Minimal (absolute deficiency predominates)	Absent; primary issue is β-cell function
Insulin/C-peptide Levels	Low or absent insulin; fasting C-peptide typically <0.3 ng/mL	Normal or elevated early (fasting C-peptide >1.0 ng/mL), with gradual decline over time	Stage-dependent—preserved or mildly reduced in early disease (stimulated C-peptide 0.8–2.0 ng/mL), progressing to reduced levels in advanced disease (stimulated C-peptide <0.6–0.8 ng/mL)	Variable by subtype—mild stable reduction (e.g., GCK-MODY) or progressive decline (e.g., HNF1A-MODY)
Glucagon Secretion	Typically preserved early, may reduce over time	Normal or exaggerated (hyperglucagonemia)	CP: Reduced or absent in advanced disease CF: Often preserved PDAC: Variable; often preserved early Post-pancreatectomy: Absent	Usually normal
Clinical Presentation	Abrupt polyuria, polydipsia, weight loss, often with ketosis	Gradual onset; fatigue, mild polyuria/polydipsia; often via complications	History of pancreatitis, steatorrhea, recurrent pain	Mild stable (GCK) or progressive hyperglycemia (HNF1A); strong family history
Associated Conditions	Other autoimmune diseases (thyroiditis, celiac, Addison’s)	Metabolic syndrome: HTN, dyslipidemia, NAFLD	Exocrine insufficiency, vitamin deficiencies, calcifications	Specific gene-related features (e.g. renal cysts in HNF1B)
Risk of Ketoacidosis	High; often presenting feature	Rare, except under stress	Possible but less common; higher hypoglycemia risk	Very low

CP, Chronic Pancreatitis; CF, Cystic Fibrosis; PDAC, Pancreatic Ductal Adenocarcinoma.

C-peptide values are provided as approximate clinical ranges. Where available, stimulated C-peptide (e.g., mixed-meal tolerance test) is preferred over fasting measurements for assessment of residual β-cell function. Interpretation should consider renal function, glycaemic status, and assay variability.

### 2.2 The Triple-Hit mechanism: integrated pathogenesis of Pancreatogenic diabetes

The ‘Triple-Hit’ paradigm conceptualizes PDM as a disorder marked by fibro-inflammatory, malabsorptive, and bihormonal deficiencies (19, 20), elucidating numerous key symptoms, such as:

- significant variation in blood glucose levels, resistant to treatment.
- unintentional loss of weight and a lack of crucial nutrients.
- unforeseen episodes of hypoglycemia from a loss of glucagon.
- increased likelihood of pancreatic malignancy.
- necessity for synchronized endocrine and exocrine restorative therapies.

The mechanistic precision offers a basis for the integration of molecular beacons, sophisticated diagnostics such as imaging and radiomics, as well as and Radiomics in the field for the application of precision medicine to this clinical condition.

**Figure 2:**
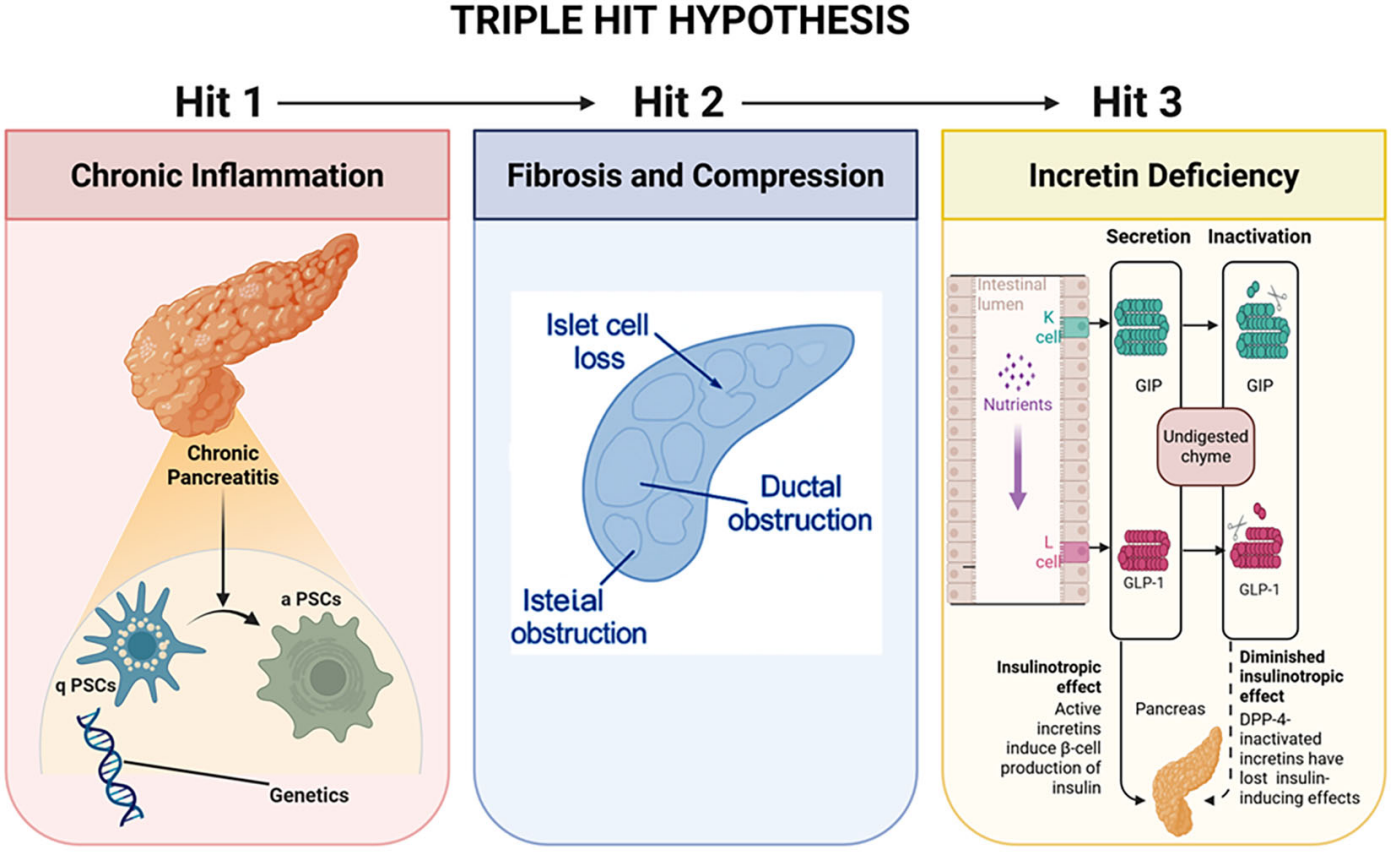
Triple-hit pathophysiologic model in inflammatory *PDM* (chronic pancreatitis–related PDM). The figure depicts three sequential mechanisms: (1) chronic inflammation activating pancreatic stellate cells, (2) fibrosis and pancreatic duct compression, and (3) incretin deficiency with reduced insulinotropic effects. Together, these processes lead to impaired β-cell function and diabetes in chronic pancreatitis. The Triple-Hit model applies primarily to inflammatory pancreatogenic diabetes associated with chronic pancreatitis and should not be extrapolated to all PDM endotypes, such as cystic fibrosis–related, paraneoplastic, or post-surgical diabetes. Created in BioRender. Babiker, R. (2025) https://BioRender.com/bh93rqu.

**Figure 2** depicts the Triple-Hit Hypothesis along with its associated metabolic consequences.

### 2.3 Clinical implications

The triple-hit paradigm shifts the understanding of PDM from an autoimmune and insulin resistant process to one that is fibro-inflammatory, malabsorptive, and bihormonal deficient (12). Knowing these factors contributes to the innovation of precision surrogates, active monitoring with customised treatment in an approach to modern diabetes (21, 22).

### 2.4 Pathophysiologic endotypes of *PDM*

The relationships between Chronic Pancreatitis, Pancreatic Ductal Adenocarcinoma, Cystic Fibrosis, and pancreatectomy are centered on pancreatogenic diabetes, yet these conditions have different driving mechanisms behind dysglycaemia. Therefore, PDM must be viewed as an umbrella definition that contains different pathophysiologic endotypes as opposed to one singular, homogeneous disease.

In PDM-CP patients, diabetes develops as a gradual process through a progressive fibro-inflammatory cascade involving the activation of pancreatic stellate cells, ductal obstruction, and the gradual decline of β and α cell mass. Among them, the Triple-Hit hypothesis- fibro-inflammatory injury, metabolic stress malabsorption, and insulin-glucagon deficiency- offers a coherent explanatory model to characterize the glycaemic instability and hypoglycaemia of advanced disease.

In contrast, the diabetes of Pancreatic Ductal Adenocarcinoma (PDM-PDAC) is often a paraneoplastic phenomenon that develops first, uncomplicated by significant pancreatic destruction. Hyperglycaemia that is mechanistically distinct from the fibrosis-driven chronic pancreatitis model is the result of factors that promote insulin resistance in the liver and β cell dysfunction, such as adrenomedullin and circulating exosomes. Insulin resistance in these patients is often more prominent, and the focal point of treatment remains oncologic management.

Cystic Fibrosis–related diabetes (PDM-CF) is defined as a genetically mediated endotype characterized by exocrine ductal blockage, islet architecture disruption, progressive loss of insulin secretion, with some retention of glucagon secretion. Given nutritional, sodium, and CFTR modulator demands, there is a need for disease tailored management strategies embedded within the broader PDM paradigm.

Post-pancreatectomy diabetes (PDM-S) involves the surgical and anatomical loss of both the endocrine and exocrine pancreas, with consequent immediate and absolute insulin deficiency and glucagon counter-regulatory loss. Unlike the inflammatory endotypes, PDM-S in isolation is not characterized by a progressive biological pathway, but rather, an abrupt shift to brittle diabetes, posing a need for considerable insulin intervention and therapy for lactose enzyme replacement.

Holding to the endotype architecture, PDM can thus act as a mechanism-based, precision class, providing a basis for functional integration while avoiding superficiality and misdirection of treatment efforts.

## 3 Diagnosis

The accurate diagnosis of PDM continues to be a diagnostic challenge in diabetology (12, 23). Therefore, a disciplined, clustered methodology that combines longitudinal patient history, revised imaging, and bespoke biochemistry is essential (24).

### 3.1 Diagnostic principles

PDM should be a diagnosis of inclusion in a certain category of patients with newly diagnosed diabetes, in their presentation with at least one of the following elements (25, 26):

- Chronic pancreatitis, pancreatic surgery, cystic fibrosis, and pancreatic cancer.
- A lean or underweight body habit, disproportionate to the degree of severity of hyperglycemia.
- An exocrine pancreatic insufficiency that presents with maldigestion symptoms, steatorrhea, bloating, and vitamin deficiencies of fat-soluble vitamins.
- Loss of glycaemic control and recurrent hypoglycemia, which is suggestive of the loss of β- cell and α-cell function.
- Uncontrolled or paradoxical responses to oral antihyperglycemic agents are observed, including metformin in conjunction with sulfonylureas.

Given that the features of T2DM can coexist particularly in overweight individuals or those with metabolic syndrome, a comprehensive assessment encompassing the structural, functional, and immunological domains provides the most accurate diagnostic reliability.

### 3.2 Evidence-based composite framework

Building on the major and minor criteria proposed by Ewald and Hardt (27) and refined by subsequent researchers, a three-tier diagnostic model provides a structured approach (**Table 4**).

**Table 4:** Three-tier diagnostic framework for PDM.

Tier	Diagnostic domain	Key parameters	Diagnostic indicator	Supporting evidence
Tier 1 – Structural Evidence	Imaging confirmation of pancreatic pathology	MRCP, EUS, CT ± elastography	Parenchymal atrophy, fibrosis, calcification, or post-surgical defect	(27)
Tier 2 – Functional Evidence	Assessment of exocrine and endocrine reserve	Fecal elastase <200 µg/g; low serum trypsinogen; reduced stimulated C-peptide (<0.8 ng/mL after MMTT)	Demonstrates exocrine insufficiency ± β-cell failure	(28)
Tier 3 – Exclusion & Biomarker Evidence	Autoimmune exclusion ± pancreatic biomarkers	Negative GAD/IA-2/ZnT8 antibodies; elevated REG1A, GP2, or miRNA signatures	Confirms non-autoimmune, pancreatic origin	(29)

## 4 Diagnostic rule

In extending previous criteria for diagnosing pancreatogenic diabetes, we suggest that, in addition to exclusion of autoimmune (type 1) diabetes, diagnosis of PDM should include both structural pancreatic pathology (Tier 1) and impaired endocrine/exocrine function (Tier 2). Some of the new predictors (Tier 3), such as REG1A and GP2 and certain radiomic features, may improve classification within certain research contexts, but are not essential for the routine diagnosis of PDM.

Demonstrable functional pancreatic impairment in PDM includes any of the following: fecal elastase-1 (FE-1) <200 µg/g, unsatisfactory coefficient of fat absorption (CFA), clinical malabsorption, or steatorrhea, any unaccountable weight loss, and deficiencies of fat-soluble vitamins. Notably, a normal FE-1 does not rule out PDM, particularly in early or patchy forms of chronic pancreatitis, in which the exocrine dysfunction may be intermittent or focal. In such instances, the presence of structural pancreatic disease (Tier 1) in combination with diabetes should prompt a diagnosis of PDM, and there should be continued functional reassessment over time.

The onset of diabetes mellitus in chronic pancreatitis is recognized as having a gradual course of progression that is multiplex in nature, with an initial stage involving preserved or mildly lowered C-peptide secretion, with driving hepatic inflammation, chronic in nature, as the primary factor in an insulin resistant state. Therefore, patients in the first tier, with clearly demonstrated diabetes and pancreatic structural damage, albeit with C-peptides that are normal or near the normal range, should not be classified as having T2DM with pancreatic calcifications as an aside. This scenario is in fact a representation of the first stages of what might be termed PDM, in which some degree of endocrine insufficiency, is present, albeit incompletely. As a continuum and not as a dichotomous phenomenon, the levels of C-peptide in the PDM diagnosis should be considered in the context of this added complexity. In the range below 0.6–0.8 ng/mL stimulated C-peptide levels, PDM is considered clinically established, with the yoke of advanced endocrine insufficiency. In contrast, ranges bordering 0.8 and 2.0 ng/mL are considered as substantiating the disease presence, especially when there is evidence of pancreatic damaging condition.

Retention of these higher levels of C-peptide do not preclude PDM when Tier 1 criteria are satisfied, but rather suggest the presence of a transitional stage in the natural history of pancreatogenic diabetes, identifying an at-risk population for future insulin deficiency, glycaemic instability, and exocrine failure meriting more intensive oversight and individualized intervention.

### 4.1 Imaging studies

Radiologic imaging continues to be essential for confirming pancreatic pathology and differentiating PDM from T1DM and T2DM. Each imaging modality offers distinct and valuable information, as summarized in **Table 5**.

**Table 5:** Imaging modalities in PDM.

Modality	Diagnostic role	Advantages	Limitations
Ultrasound (US)	Detects ductal dilation, calcifications, pseudocysts useful for chronic pancreatitis.	Widely available, inexpensive, non-invasive.	Operator-dependent, limited for early disease.
Magnetic Resonance Cholangiopancreatography (MRCP)	Defines ductal and parenchymal anatomy; detects subtle fibrosis and strictures.	High soft-tissue contrast, non-invasive, no radiation.	Costly, limited access, contraindicated in metal implants.
Endoscopic Ultrasound (EUS)	Detects early parenchymal changes, small neoplasms; enables tissue sampling.	High resolution; permits FNA biopsy.	Invasive, requires sedation; small risk of pancreatitis.
Elastography	Quantifies tissue stiffness, staging fibrosis, differentiates benign from malignant lesions.	Provides objective fibrosis assessment.	Adjunctive tool; requires technical expertise.

**Table 5**. Summary of imaging modalities used to diagnose PDM and associated pancreatic disorders.

### 4.2 Laboratory and functional testing

Laboratory tests complement imaging by quantifying both the endocrine and exocrine pancreas reserve. Each approach offers specific information on exocrine and endocrine pancreatic functions, autoimmunity, and nutritional status. The diagnosis and differential diagnosis of Pancreatogenic DM, along with the assessment of the degree of pancreatic involvement, usually require several tests to be performed (**Table 6**).

**Table 6:** Summary of key laboratory and functional tests in the diagnostic workup of PDM.

Test	Purpose	Advantages	Limitations
Fecal elastase-1	Confirms exocrine insufficiency (<200 μg/g).	Non-invasive, unaffected by enzyme therapy.	False negatives in diarrhea.
Serum trypsinogen	Screens exocrine function (esp. in children).	Useful in pediatric settings.	Low sensitivity in adults.
C-peptide (fasting/stimulated)	Assesses residual β-cell function.	Differentiates T1DM, T2DM, PDM.	Influenced by renal function and glucose levels.
Autoantibodies (GAD, IA-2, ZnT8)	Exclude autoimmune diabetes.	Clarifies etiology.	Negative in both T2DM and PDM.
Oral Glucose Tolerance Test (OGTT)	Detects early β-cell failure.	Sensitive and widely available.	Time-consuming.
Micronutrient Panels (A, D, E, K, B12)	Detect malabsorption and nutritional risk.	Complements exocrine assessment.	Indirect indicator.

### 4.3 Practical diagnostic algorithm

**Figure 3:**
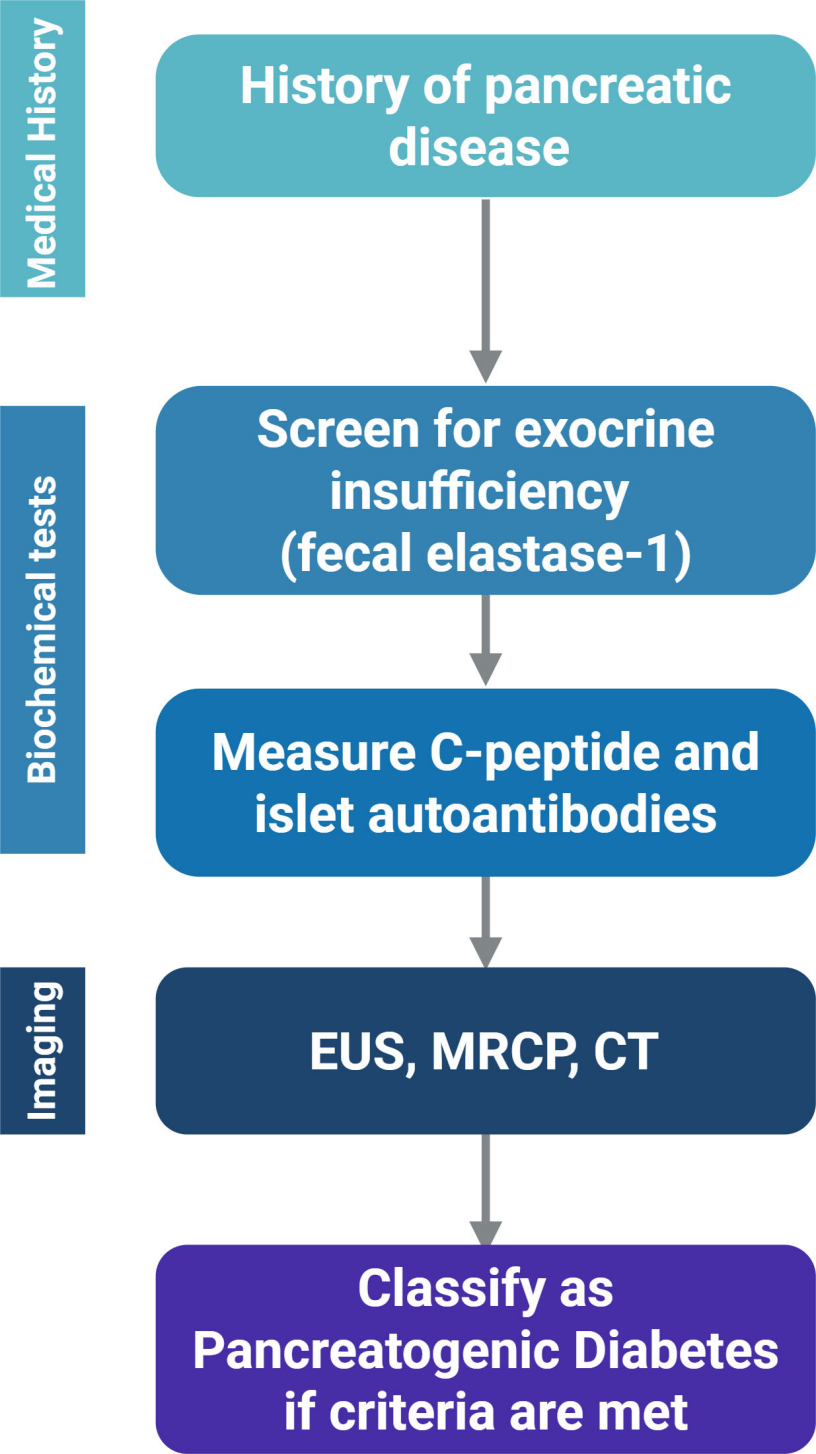
Presents a stepwise diagnostic framework for PDM. Created in BioRender. Babiker, R. (2025) https://BioRender.com/9hxthmh.

The finalisation of a diagnosis of PDM requires triangulation of clinical signs, biochemical data, and imaging studies (30). A stepwise approach to diagnosis is suggested, as shown in **Figure 3**.

1. Identify any history or imaging evidence that indicates pancreatic disease.
2. Assess exocrine pancreatic function by measuring faecal elastase-1 and/or serum trypsinogen.
3. Measure C-peptide levels and pancreatic autoantibodies as a means to evaluate endocrine function and to rule out autoimmune involvement.
4. It is advisable to use MRCP or Endoscopic Ultrasound (EUS) for confirmation.

### 4.4 Clinical red flags for misclassification

- Failure to perform pancreatic imaging in patients with new onset diabetes mellitus after the age of 50.
- The absence of faecal elastase testing in the diagnosis and management of chronic pancreatitis.
- Insulin dependence in pancreatogenic diabetes is frequently misinterpreted, but the direction of misclassification varies according to patient phenotype. In older or overweight individuals, who represent the predominant demographic for chronic pancreatitis– and pancreatic cancer–associated diabetes, insulin requirement is commonly assumed to reflect advanced Type 2 diabetes with failure of oral therapy, leading to under-recognition of pancreatic disease and delayed initiation of enzyme replacement, nutritional support, and cancer surveillance. Conversely, in younger or lean patients, including those with hereditary pancreatitis, cystic fibrosis, or post-pancreatectomy states, early insulin dependence may mimic Type 1 diabetes, despite the absence of autoimmune markers. Recognition of these dual misclassification pathways highlights the need for routine consideration of pancreatic etiology in insulin-requiring diabetes, particularly when accompanied by gastrointestinal symptoms, weight loss, or imaging abnormalities.

**Figure 4:**
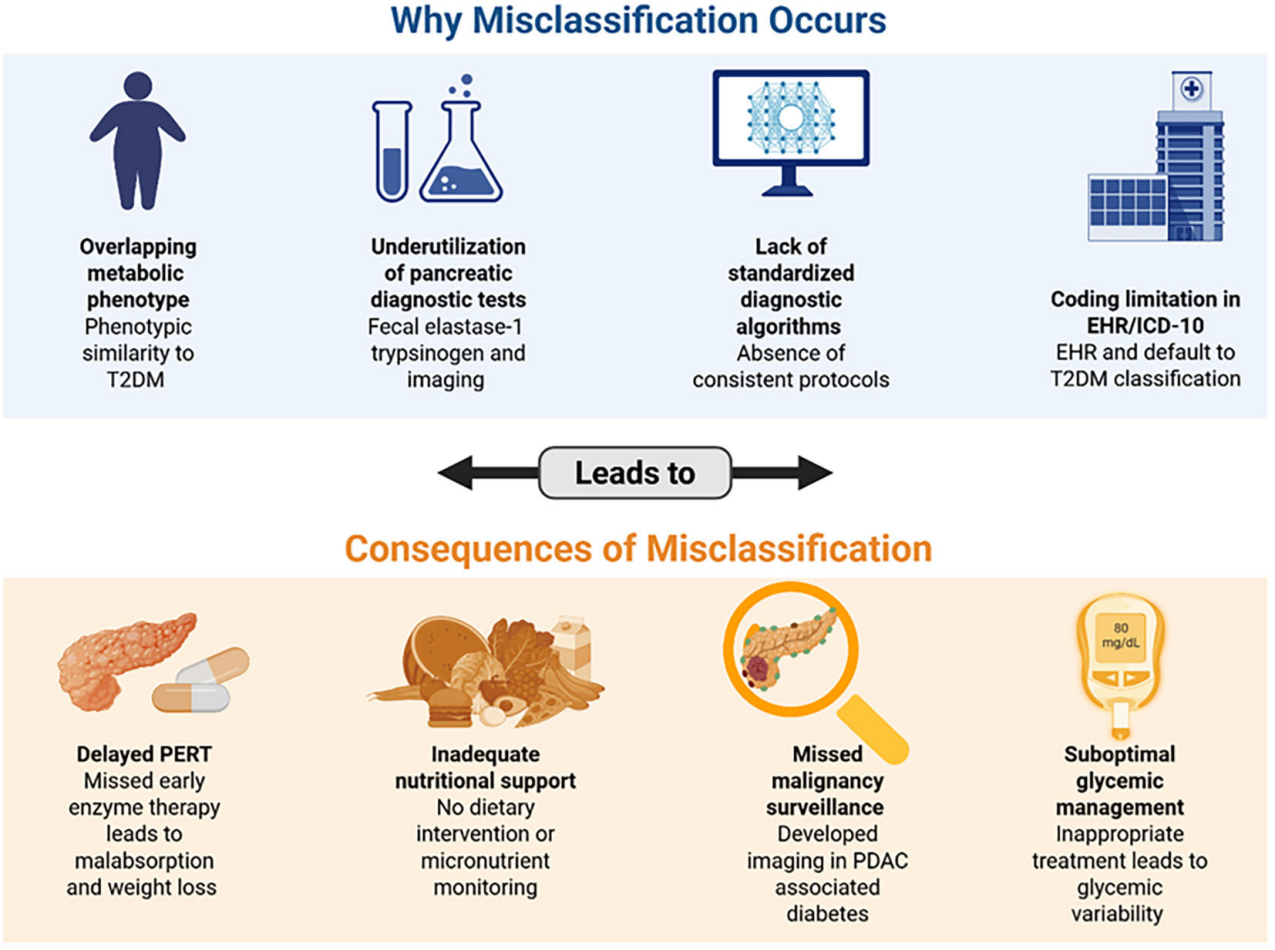
Depicts the primary factors contributing to the misclassification of PDM, along with the downstream implications such as diagnostic delays, inappropriate treatment decisions, and increased risk of disease progression. Created in BioRender. Babiker, R. (2025) https://BioRender.com/vqaw9qw.

Identifying these red flags can prevent therapeutic misdirection and enhance patient outcomes by facilitating early pancreatic assessment and the initiation of enzyme replacement therapy. **Figure 4** depicts the factors contributing to the misclassification of PDM and its subsequent consequences.

## 5 Glycaemic monitoring: beyond HbA1c

Monitoring glycaemic control in PDM requires an individualised approach. In this group of patients, as with other long-term control measurements, HbA1c is often not useful due to multiple confounders, particularly autoimmune type inflammatory conditions (31, 32).

### 5.1 Limitations of using HbA1c

- Although HbA1c provides an estimate of mean glycaemic levels over the last two to three months, there are a number of pathophysiological factors in PDM that negatively impact the precision of the measure (32, 33).
- HbA1c interpretation in PDM is further complicated by hematological abnormalities related to malnutrition and chronic disease. Iron deficiency anemia typically prolongs red blood cell lifespan and increases cumulative glycation exposure, leading to a falsely elevated HbA1c, whereas conditions associated with shortened erythrocyte survival—such as hemolysis or acute blood loss—result in falsely lowered HbA1c values. Vitamin B12 and folate deficiencies (megaloblastic anemia) interfere with effective erythropoiesis and are associated with reduced red cell turnover, often producing falsely elevated HbA1c levels, although variability may occur depending on disease severity and treatment status. These opposing biases further limit the reliability of HbA1c as a sole marker of glycaemic control in patients with PDM. These hematological confounders reinforce the need to incorporate alternative glycaemic markers and continuous glucose monitoring metrics when assessing glycaemic control in PDM.
- Erratic hyper and hypoglycemia due to a combined deficiency of insulin and glucagon, malabsorption, and inconsistent calorie intake obscure actual variability.
- In patients with exocrine insufficiency or anaemia, there can be a significant difference between capillary glucose measurements and actual glucose levels, which can lead to erroneous adjustments of insulin dosage.

### 5.2 Alternative glycemic biomarkers and monitoring tools

Integrating biochemical and sensor-based tools ensures dynamic assessment of glycemia in PDM. (**Table 7**).

**Table 7:** Alternative glycemic biomarkers and their clinical utility in PDM.

Marker	Monitoring window	Clinical utility	Supporting evidence/reference
Fructosamine	2–3 weeks	Reflects short-term glycemia; useful when therapy is being titrated or HbA1c is unreliable. Less influenced by hemoglobinopathies.	(34)
1,5-Anhydroglucitol (1,5-AG)	1–2 weeks	Sensitive indicator of postprandial excursions; declines after glucose surges > 180 mg/dL.	(35)
Continuous Glucose Monitoring (CGM)	Real time (24 h)	Quantifies glycemic variability and nocturnal hypoglycemia; enables calculation of *Time-in-Range (TIR)* and supports insulin titration.	(36)
Flash Glucose Monitoring (FGM)	Intermittent sensor-based	Provides trend data at lower cost; suitable for routine outpatient follow-up in resource-limited settings.	(37)

### 5.3 Practical clinical strategy

- A multi-faceted approach is suggested for chronic PDM to understand the complexity of both short-term and long-term glycemic control.
- Incorporating continuous glucose monitoring (CGM) or flash glucose monitoring (FGM) alongside short-term therapeutic evaluation via fructosamine levels helps assess daily glycemic variability.
- When insulin or enzyme therapy is titrated, focus on Time-in-Range (TIR) and glycemic variability indices as opposed to using an isolated HbA1c.
- 1,5-anhydroglucitol (1,5-AG) should be used only when there is a suspicion of postprandial hyperglycemia, even with normal HbA1c or fructosamine levels.

### 5.4 Current guidelines and future perspectives

The ADA and EASD guidelines do not specifically suggest alternative markers for PDM. Consequently, these tools ought to be used in conjunction with, not in lieu of, existing clinical evaluations until specific validation studies are performed. Future exploration should center on combining CGM metrics and biochemical markers with artificial intelligence in order to customize glycemic targets for individuals with PDM.

Furthermore, as there are no specific validation studies to support the use of additional clinical tools for PDM, these tools should only be used to assist clinicians in the absence of other benchmark tools.

## 6 Therapeutic management of PDM

Management strategies should be tailored to the underlying PDM endotype.A multidisciplinary approach that includes endocrinologists, gastroenterologists, dietitians, and diabetes educators is essential to achieve optimal outcomes (38) (**Table 8**).

**Table 8:** Provides a summary of the primary insulin and adjunctive therapies employed in the management of PDM.

Therapy class	Mechanism of action	Clinical evidence in PDM	Key limitations
Basal–Bolus Insulin	Restores physiologic insulin pattern	Standard of care; improves glycemic stability	Hypoglycemia risk; requires monitoring
CSII (Insulin Pump)	Continuous infusion with real-time glucose feedback	Effective in brittle diabetes; improves TIR	Cost, training, access
Premixed Insulin	Simplified basal + bolus in one injection	Useful for elderly or adherence issues	Less individualized; post-meal variability
Metformin	Improves hepatic insulin sensitivity	Limited role; consider if overweight	GI intolerance; contraindicated in malnourished
Sulfonylureas	Stimulate residual β-cells	Ineffective in advanced disease	High hypoglycemia risk
DPP-4 Inhibitors	Prolong endogenous GLP-1 activity	Minimal theoretical benefit	Sparse data; limited efficacy
GLP-1 Agonists	Enhance insulin, suppress glucagon	Not recommended in pancreatitis	Poor tolerance; GI effects
Dual GLP-1/GIP Agonists	Dual incretin stimulation	Investigational	Unknown safety; avoid in exocrine disease

### 6.1 Insulin therapy

Absolute insulin deficiency is the main characteristic of PDM that makes insulin replacement the foundation of its treatment (39).

#### 6.1.1 Principles of insulin therapy

1. The most flexible option from a physiological point of view is the basal-bolus regimen, which comprises basal insulins of long-acting formulations such as glargine and degludec, and rapid-acting insulins at meal-time.
2. For patients with unstable glycemia and those with frequent hypoglyzemias, continuous subcutaneous insulin infusion (CSII) or insulin pump therapy is the preferred option.
3. Instead of HbA1c levels, insulin dose adjustments should be made to ensure that the Time-in-Range (TIR) level improves, as this metric is derived from continuous glucose monitoring (CGM).
4. It is necessary to educate patients on the prevention of hypoglycemia, especially because the function of α-cells, which are responsible for counter-regulatory control, is lost.

### 6.2 Non-insulin agents

1. Use of metformin should be considered for patients with insulin resistance and those who are overweight, although GI side effects that affect tolerability are often considered (40).
2. There is a risk of hypoglycemia and sulfonylureas are largely ineffective (41).
3. Their use is limited to the experimental realm, as there is little to no efficacy from DPP-4 inhibitors, GLP-1 receptor agonists and dual GLP-1 and GIP agonists like Tirzepatide due to the already deficient secretion of incretins (42). The safety data concerning their use in pancreatogenic disease remain unavailable (43).

### 6.3 Pancreatic enzyme replacement therapy

Pancreatic Enzyme Replacement Therapy (PERT) substantially aids in the treatment of Exocrine Pancreatic Insufficiency (EPI) and indirectly helps improve the control of hyperglycemia (44, 45).

- Mechanisms behind the benefits include the following.
- Improvement of nutrient absorption and protection against malnutrition.
- Decreased post meal hyperglycemia through the regulation of GLP-1 and GIP (two of the incretins).
- Improvement of metabolic control and quality of life.

Dosing and Administration

- Start with 40,000 to 50,000 units of lipase per main meal and 10,000 to 25,000 units per snack.
- Use of enteric coated products is highly recommended. If persistent symptoms occur, the addition of a proton pump inhibitor (PPI) may reduce symptoms.
- For maximum effectiveness, medications should be taken at the beginning of the meal and continue throughout the meal.

Monitoring response

- Observe changes in stool patterns, body weight, and levels of fat-soluble vitamins (A, D, E, and K).
- In the case that symptoms and/or stool fat levels remain high, dosage adjustments may be required.

#### 6.3.1 Expected outcomes

Pancreatic enzyme replacement therapy (PERT) improves fat digestion and dietary fat absorption, overall nutrition, and likely reduces glycaemic variability. If malnutrition continues despite appropriate dosing, a consultation with a dietitian is suggested to evaluate and modify enzyme therapy and total dietary energy.

### 6.4 Nutritional and micronutrient management

Nutritional rehabilitation is crucial to reverse malnutrition and achieve metabolic stabilization (46, 47).

Core Dietary Recommendations:

- A daily caloric intake of 25–30 kcal/kg should be recommended, consisting of 50-60% carbohydrates, and protein and fat as per individual requirement.
- Diets that are very low in fat should be avoided, as they can worsen steatorrhea.
- High protein foods and medium-chain triglycerides (MCTs) fats should be eaten more, and frequent small meals are recommended.
- To facilitate reduction in disease progression and risk of carcinoma, abstinence from alcohol and smoking is necessary.

Vitamin and Micronutrient supplementation

- Routine replacement of fat-soluble vitamins A, D, E, and K and vitamin B12 should be recommended.
- 25-hydroxy vitamin D levels should be monitored every 6–12 months and DEXA scans should be performed every 2 years for bone health.
- Zinc and magnesium deficiencies that may cause poor glycaemic control should be addressed.

### 6.5 Surveillance for complications

Patients with PDM represent a clinically important population with an elevated association between pancreatic disease and pancreatic ductal adenocarcinoma (PDAC) (47). However, pancreatic imaging strategies must differentiate between initial diagnostic screening and long-term surveillance, as these approaches carry different clinical and health-economic implications.

Diagnostic Evaluation. In previously healthy individuals over 50 who experience the sudden onset of diabetes, and in those patients with newly diagnosed chronic pancreatitis who also present with weight loss, glycemic decompensation, abdominal pain, and other abdominal symptoms, a one-time assessment using high-resolution pancreatic imaging (MRCP or EUS) is warranted to rule out the possibility of occult PDAC. The approach is clinically justified based on studies which have shown that diabetes often precedes other clinical signs of pancreatic malignancy.

Long term surveillance. In stark contrast to the previous population, the routine practice of annual surveillance imaging is not applicable to all patients who have PDM, or chronic pancreatitis. There is a consensus amongst the clinical and scientific community, including recommendations by the International Association of the Pancreas (CAPS) consortium, that a chronic surveillance program is indicated for specific population cohorts, including but not limited to individuals with hereditary pancreatitis, patients with strong familial pancreatic cancer syndrome, and patients with uncertain diagnostic criteria for pancreatic cystic lesions. Such individuals are subject to risk stratification to determine the merit of periodic imaging with EUS or MRI/MRCP.

While the heightened lifetime risk of PDAC in pancreatogenic diabetes raises the possibility that earlier or more frequent surveillance could improve outcomes, such an approach currently exceeds existing guideline recommendations and would require formal evaluation of cost-effectiveness, diagnostic yield, and patient-centered outcomes before broad implementation. Accordingly, we propose that intensified surveillance strategies in PDM be regarded as hypothesis-generating and investigational, rather than standard of care, pending prospective validation.

#### 6.5.1 Pancreatic cancer surveillance

- New diabetes diagnosis after age 50, or new chronic pancreatitis, requires a yearly evaluation of EUS or MRCP.
- Genetic screening for PRSS1, CFTR and SPINK1 gene defects will help identify people who may be at increased risk.
- CA 19–9 and other biomarkers may be useful, although their specificity is low (47).

#### 6.5.2 Skeletal health

Skeletal complications in PDM arise primarily from chronic inflammation, malabsorption, and vitamin D deficiency, and their evaluation and management are consistent with existing gastroenterology and endocrinology guidelines.

Vitamin D deficiency is common in PDM due to fat malabsorption associated with exocrine pancreatic insufficiency, and effective correction requires optimization of pancreatic enzyme replacement therapy (PERT), as oral cholecalciferol is fat-soluble and dependent on adequate pancreatic lipase for absorption. In the absence of sufficient enzyme replacement, vitamin D supplementation may be ineffective despite appropriate dosing, and patients with severe or refractory malabsorption may require alternative approaches such as water-miscible or liquid formulations, calcidiol (25-hydroxyvitamin D), or supervised non-oral therapies (48). The increased risk of osteoporosis in PDM reflects the combined effects of chronic inflammation, vitamin D deficiency, and malnutrition; therefore, management should include integrated nutritional assessment, calcium supplementation (1,000–1,200 mg/day), vitamin D supplementation (800–1,000 IU/day), and biannual bone mineral density assessment using dual-energy X-ray absorptiometry (DEXA), rather than isolated micronutrient replacement.

#### 6.5.3 Gastrointestinal complications

Gastrointestinal complications in PDM reflect established consequences of exocrine pancreatic insufficiency and are managed according to current standard clinical pathways, rather than disease-specific surveillance protocols (49).

• Persistent steatorrhea or unexplained weight loss in people with PERT may be indicative of small intestinal bacterial overgrowth (SIBO) or celiac disease and should therefore be referred for a gastroenterology assessment.

### 6.6 Psychosocial and multidisciplinary care

- PDM substantially affects quality of life due to recurrent pain, dietary restrictions, and anxiety related to hypoglycemia.
- Incorporate psychological support with organised educational activities related to self-monitoring and nutrition.
- Regular contact with a dietitian and diabetes educator improves patient compliance and control of blood sugar level.
- It has been shown that the multidisciplinary approach reduces hospitalisation and increases patient satisfaction.

### 6.7 Pain management and opioid stewardship

The management of pain is correlated to the metabolic stability of PDM and is especially salient when considering the case of chronic pancreatitis. Factors such as Persistent pancreatic pain leads to the chronic activation of the sympathetic nervous system and the hypothalamic–pituitary–adrenal axis, which in turn leads to an increase of catecholamines and cortisol. Such substances would further antagonize the action of insulin and subsequently increase gluconeogenesis of the liver and worsen the glycaemic variability. The lack of effective pain management, therefore, has the potential to amplify insulin resistance, unstable glycaemic control, and the resultant increase in insulin dosage.

Improved systems of pain management in patients with PDM must be viewed as more than a means of improving life but also as a means of improving glycaemic control. The primary modalities which should be incorporated as a first-line strategy when possible include, but are not limited to, non-opioid analgesics, the optimization of pancreatic enzymes, available neuromodulators, endoscopic or interventional pain procedures, and psychological pain coping techniques. The planned and careful use of opioids, when necessary, is warranted which should also include control of their negative side effects which include poor control of gastrointestinal motility, supply of nutrients, awareness of hypoglycaemia, and metabolic control. To achieve the predicted metabolic and functional results in this subgroup of the population, there needs to be close collaboration between endocrinologists, gastroenterologists, pain medicine specialists and mental health professionals.

**Figure 5:**
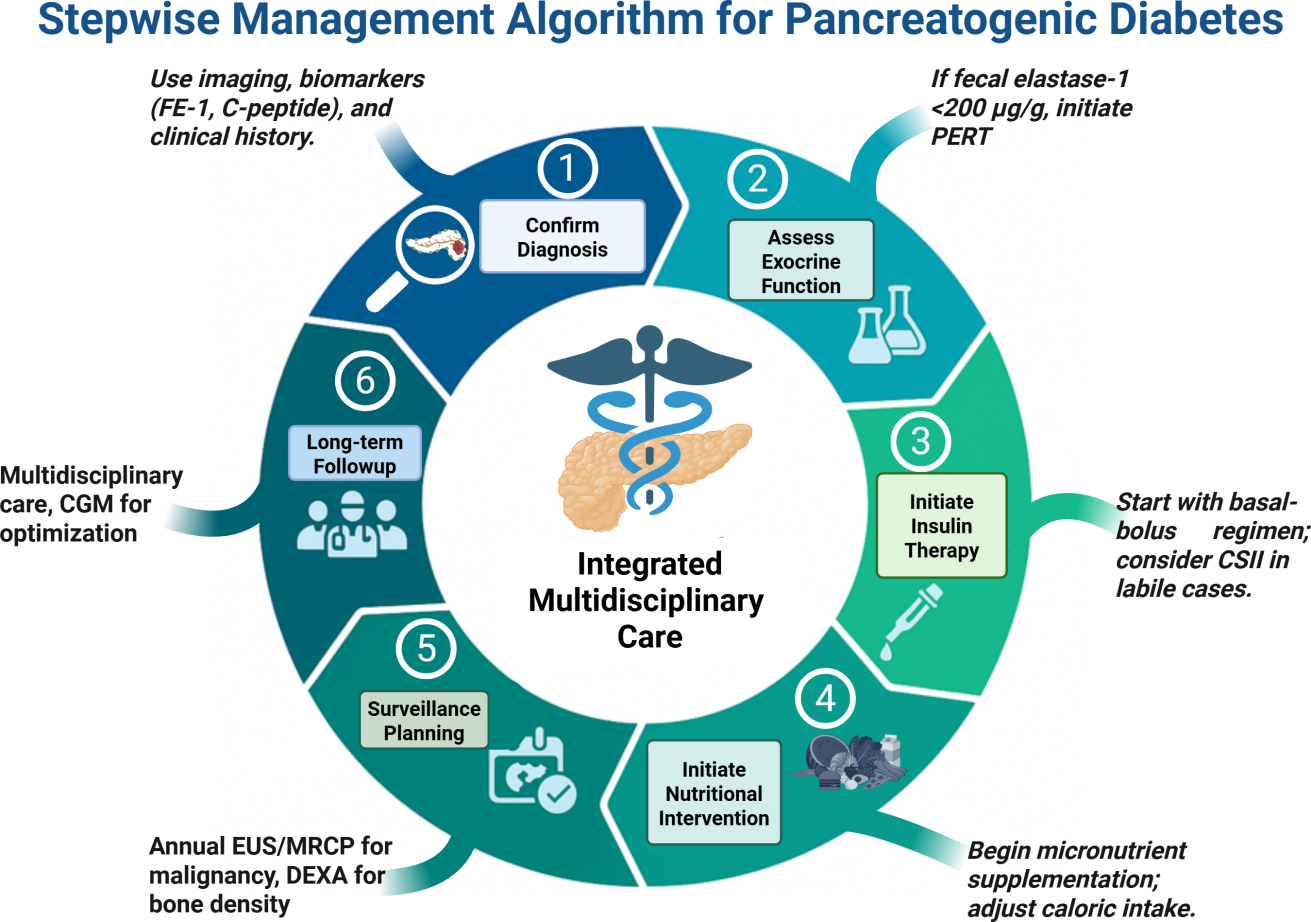
Stepwise management algorithm for PDM, illustrating the sequential clinical decision-making process from initial assessment to advanced therapeutic interventions. Created in BioRender. Babiker, R. (2025) https://BioRender.com/45a1dce.

**Figure 5** presents the stepwise management algorithm for PDM.

## 7 Prognosis and complications

PDM has a 2.5-3-fold higher burden and mortality rate than Type 2 Diabetes Mellitus (50).

### 7.1 Major complications and clinical management

**Table 9** provides the key systemic complications of pancreatogenic diabetes mellitus (PDM), their pathophysiological basis, clinical presentation, and treatment. Their complications are the result of a complex effect of endocrine and exocrine pancreatic dysfunction, long-term inflammation, and malnutrition. Nutritional deficiencies, musculoskeletal degradation, and elevated risk of fracture are accompanied with metabolic imbalances, including severe hypoglycemia and electrolyte imbalance. Long-term risks such as pancreatic cancer and cardiovascular disease and microvascular disease have also been highlighted in the table. All in all, it highlights the importance of the multidisciplinary approach that pays attention to the metabolic control, nutritional recovery, and frequent monitoring of the systemic complications.

**Table 9:** Systemic complications in PDM.

Complication	Underlying mechanism	Clinical features	Management approach
Severe Hypoglycemia	Loss of α- and β-cell mass impairs counterregulation and hepatic glucose output.	Neuroglycopenia, confusion, seizures, coma.	Frequent glucose monitoring; cautious insulin titration; patient education.
Fat-Soluble Vitamin Deficiencies (A, D, E, K)	Exocrine insufficiency → fat malabsorption and impaired micelle formation.	Night blindness, osteomalacia, neuropathy, coagulopathy.	PERT, vitamin replacement, and nutritional monitoring.
Sarcopenia and Cachexia	Chronic inflammation and malnutrition reduce muscle mass and IGF-1 activity.	Muscle wasting, frailty, poor mobility, higher fracture risk.	Nutritional rehabilitation, exercise, consider anabolic therapy.
Osteoporosis and Skeletal Fragility	Vitamin D and calcium deficiency; systemic inflammation.	Vertebral and femoral neck fractures; delayed healing.	DEXA every 2 years; calcium and vitamin D; bisphosphonates if indicated.
Dehydration and Electrolyte Imbalance	Osmotic diuresis and steatorrhea cause loss of Na, K, Mg.	Weakness, arrhythmias, hypotension.	Oral/IV rehydration, electrolyte repletion.
Pancreatic Cancer (PDAC)	Chronic inflammation promotes dysplasia; PDAC may induce diabetes.	New-onset diabetes >50 years, weight loss, abdominal pain.	Annual EUS/MRCP in high-risk; oncology referral; CA 19–9 adjunct.
Cardiovascular and Microvascular Disease	Oxidative stress and micronutrient deficiency damage endothelium.	Atherosclerosis, retinopathy, nephropathy, neuropathy.	Blood pressure and lipid control; vitamin repletion; foot/eye screening.

### 7.2 Predictors of poor outcomes

**Table 10** describes some of the main prognostic determinants that affect the results in pancreatogenic diabetes mellitus (PDM). It emphasises the importance of the nutritional status, progression of pancreatic disease and compliance with enzyme, insulin, and nutritional treatments in the morbidity and mortality. The table also highlights the issue of pancreatic ductal adenocarcinoma which needs to be observed carefully in patients with recently developed or progressive diabetes.

**Table 10:** Prognostic determinants in PDM.

Prognostic factor	Clinical implication	Key references
Persistent malnutrition (BMI <18.5 kg/m²)	Independent predictor of mortality; correlates with low fecal elastase and albumin.	(51)
Recurrent pancreatitis or progressive fibrosis	Accelerates endocrine–exocrine failure and brittle glycemia.	(52)
Absence of enzyme replacement	Increases hospitalization and mortality.	(53)
New-onset or worsening diabetes in chronic pancreatitis	May signal underlying PDAC.	(54)
Non-adherence to insulin or nutrition therapy	Predicts recurrent hypoglycemia and hospital readmissions.	(55)

### 7.3 Emerging predictive tools

Novel innovations in radiology and machine learning have made it possible to quantitatively measure pancreatic fibrosis and β-cell reserve.

- With an accuracy of 85%, texture analysis from computed tomography (CT) and magnetic resonance imaging (MRI) is able to predict post-pancreatitis diabetes (56).
- Predictive of case identification and risk stratification automation in the future will be artificial intelligence (AI) classifiers that synergise imaging, C-peptide and elastase, and faecal measurements.

The use of these technologies in longitudinal care pathways makes it possible to detect individuals at high risk early, resulting in personalised surveillance strategies (**Table 11**).

**Table 11:** Clinical and research-stage translational status of diagnostic tools.

Translational status	Examples
Current clinical use	MRCP, EUS, biochemical profiling
Research-only tools	Radiomics, miRNA panels, REG1A, GP2
Future potential	Integrated AI-driven platforms

Despite growing interest in radiomics and artificial intelligence–based image analysis for pancreatogenic diabetes, several critical barriers currently limit their routine clinical adoption. Radiomic texture features derived from computed tomography or magnetic resonance imaging—such as entropy, kurtosis, and higher-order texture matrices—are highly sensitive to image acquisition parameters, including scanner manufacturer, slice thickness, contrast phase, and reconstruction algorithms, resulting in limited reproducibility and reduced performance during external validation. The absence of standardized pancreatic imaging protocols and harmonized feature-extraction pipelines therefore precludes the incorporation of radiomics into universally applicable diagnostic frameworks at present. Accordingly, within the proposed diagnostic model, Tier 1 structural assessment relies on established clinical imaging modalities such as MRCP and endoscopic ultrasound, whereas quantitative radiomics and AI-driven texture analysis are positioned within Tier 3 as investigational tools intended for future integration. Similarly, emerging molecular approaches—including circulating microRNA panels and candidate biomarkers such as REG1A and GP2—remain largely research-oriented, supported predominantly by retrospective or small cohort studies and lacking robust prospective validation. Beyond technical and evidentiary limitations, the absence of large-scale validation, cost-effectiveness analyses, and implementation data—particularly in low-resource settings where pancreatogenic diabetes may be under-recognized yet prevalent—represents a major barrier to near-term translation. Collectively, these considerations underscore that AI-driven imaging and molecular biomarkers should currently be regarded as hypothesis-generating and risk-stratification tools, with future clinical integration contingent upon rigorous multicenter validation, standardization, and explicit attention to health-system feasibility and equity.

### 7.4 Long-term follow-up recommendations

Organising annual follow-up plans is vital for effective oversight.

- A two-fold review of endocrinology with nutrition is conducted biannually to optimise insulin and enzyme therapy.
- Vitamin D and some elemental micronutrients should be tested biannually with a DEXA scan to assess bone density every two to three years.
- Chronic pancreatitis lasts more than a decade and a history of familial PDAC is recommended to receive annual pancreatic imaging by EUS or MRI.
- Routine screening for diabetic complications—including retinal, renal, and foot exams should follow the ADA 2024 standards.
- Adherence and survival are affected by a psychological review for depression, anxiety, and chronic pain.

The prognosis of PDM depends primarily on the degree of pancreatic disease, nutritional status, and whether or not integrated care is timely. Obtaining an accurate diagnosis, screening for foresight complications, and managing through many specialities are essential to improve the chances of long-term survival with a good quality of life.

## 8 Future directions and research gaps

Redefining PDM provides a new framework to integrate molecular biology, imaging, and digital medicine into clinical endocrinology. However, significant evidence gaps and implementation challenges must be addressed before it is recognised worldwide.

### 8.1 Standardization and digital integration in diagnosis

Recent estimates of prevalence and misclassification have resulted from differing criteria for the diagnosis of PDM. This highlights the need to establish international criteria to harmonise clinical practices, initiate new investigations, and coding of the disorder.

- Future consensus protocols should address the larger questions of improving scalability, decentralisation, and security.
- Specify the necessary diagnosis levels to include structural, functional, and exclusion criteria.
- Identify how REG1A, GP2 biomarkers, and particular microRNAs may be useful for early diagnosis.
- Propose harmonised diagnostic imaging protocols that include MRCP, EUS, and elastography.
- Propose the new ICD codes to clarify PDM as distinct from secondary and/or other unspecified diabetes.

On the other hand, diagnostic accuracy and reproducibility are being improved using Artificial Intelligence (AI) and Radiomics analytiX.

- Non-invasively, radiomic algorithms have the ability to assess the architecture of the pancreatic and the extent of fibrosis to predict the endogenous β-cell reserve.
- PDM and T2DM can be differentiated by the use of machine learning algorithms that integrate various data points from imaging, faecal elastase, and C-peptide.
- Some models are capable of predicting glycaemic variability and helping with the appropriate titration of insulin.

To ensure that the benefits of technological progress are made available for uniform practices to be implemented worldwide, the collaboration of diverse networks for data sharing is the most efficient way of training and validating algorithms devised for different imaging technologies.

### 8.2 Molecular and genetic insights

Genomic and transcriptomic studies are beginning to elucidate the molecular pathways that link chronic inflammation to β-cell failure and carcinogenesis. Future research priorities include.

- Determining specific gene alterations that act as markers of progression such as SPINK1, PRSS1 and CFTR.
- Characterisation of specific methylation patterns associated with the insulin gene and the GLP-1 receptor.
- Create microRNA and exosome-based biomarkers to design liquid biopsy platforms that can increase early disease diagnosis and follow-up.

Translational studies involving the integration of molecular data with imaging and clinical biochemistry can facilitate the application of personalised medicine to PDM.

### 8.3 Therapeutic innovation and clinical trials

The specific clinical trial for PDM is scarce, Research focus must include:

- Identify specific insulin delivery technologies, such as closed-loop pumps, and evaluate their impact together with AI for adaptive dosing.
- Using standard endpoints to assess the metabolic impact of PERT (pancreatic enzyme replacement therapy) and its optimisation.
- Search and describe novel therapeutic agents such as dual agonists, modulators of the gut microbiome, and pan-fibrotic agents directed to pancreatic injury.
- Conduct long-term studies of the results to determine the main factors of mortality and the variables that alter the associated cancer risk.
- These studies would benefit from International registry for the harmonisation of data and multicenter collaboration.

### 8.4 Policy, education, and global collaboration

Recognising PDM in clinical practice requires policy and educational reforms.

- Including PDM as a distinct category in the WHO and ADA classifications will improve disease coding, resource allocation, and patient awareness.
- Integrating curriculum in medical schools and speciality training improves early recognition and reduces misdiagnosis.
- Establishing regional PDM registries in collaboration with academic consortiums (e.g. PancreasNet, ADA-EASD) can improve epidemiological surveillance and research.
- Patient advocacy programmes improve adherence, reduce stigma of malnutrition, and encourage multidisciplinary care.

### 8.5 The road ahead: advancing precision endocrinology

Recognition of PDM marks a shift from phenotype-based classification to mechanism-based precision endocrinology. Future efforts should focus on:

- Validating composite diagnostic algorithms that integrate clinical, imaging, and molecular data.
- Integrating AI-driven decision-support tools into clinical workflows improves healthcare delivery.
- Linking registry data with national cancer and metabolic databases enables effective outcome tracking.

The proposed classification of PDM should be viewed primarily as a health-systems and care-delivery intervention, intended to correct persistent misclassification and under-treatment, rather than as an attempt to expand numerical diabetes taxonomy.

In summary, advancing from conceptual recognition to formal inclusion of PDM in global diabetes frameworks requires standardised definitions, digital innovation, and cross-disciplinary collaboration. Integrating these elements will ensure that PDM is recognised as a distinct, measurable, and treatable condition.

## 9 Limitations

This review coalesces the literature across various domains, but is also governed by multiple limitations.

- There is an extensive body of literature on PDM; however, this literature is heterogeneous and, in some cases, characterised by retrospective studies, inconsistent diagnostic criteria, and other methodological shortcomings.
- In analyses of this nature, the absence of specific ICD categorisation and standardised diagnostic criteria makes it difficult to disentangle T1DM or T2DM cases, resulting in misclassification and in some scenarios even diagnostic overlap.
- There is a relative lack of prospective or randomised clinical studies in cohorts of PDM on specific interventions, such as advanced strategies to optimise enzyme replacement therapy, emerging insulin delivery systems or dual incretin agonists.
- The inclusion of AI-enhanced radiomics and molecular pathways is highly inventive; however, the majority of such innovations have not yet reached a clinical setting.
- This review reflects the limitations of the available literature up to June 2025; future multicenter trials and standardised registries are expected to further refine diagnostic and therapeutic paradigms.

Despite these limitations, the review lays the foundation of emerging molecular understanding of endocrinology to stimulate clinical recognition and encourage a more comprehensive approach to research.

## 10 Conclusion

Pancreatogenic diabetes remains under-recognised, frequently misclassified, and clinically underserved despite its substantial impact on morbidity and mortality. In this review, we propose Pancreatogenic Diabetes Mellitus (PDM) as a mechanistically expanded and clinically operational framework that builds on, but moves beyond, the traditional concept of Type 3c diabetes. By integrating contemporary insights into fibro-inflammatory pancreatic injury, endocrine–exocrine cross-talk, dynamic β-cell dysfunction, and disease-specific endotypes, PDM provides a structured approach to diagnosis and management that better reflects real-world clinical complexity.

The proposed tiered diagnostic framework, emphasis on stage-dependent endocrine failure, and recognition of distinct PDM endotypes aim to improve diagnostic accuracy, reduce misclassification, and support individualized metabolic and nutritional care. Although emerging tools such as radiomics, molecular biomarkers, and artificial intelligence offer promise for future refinement, their current role remains investigational and depends on prospective validation, standardisation, and equitable implementation.

Ultimately, the adoption of a unified PDM framework has the potential to improve clinical recognition, guide multidisciplinary management, and stimulate targeted research efforts. Future studies should focus on validating diagnostic thresholds, defining endotype-specific therapeutic strategies, and evaluating cost-effective approaches applicable across diverse health-care settings.
