# Correction: Redefining pancreatogenic diabetes mellitus through molecular, imaging, and AI-driven evidence

**DOI:** 10.3389/fendo.2026.1810879

**Published:** 2026-04-08

**Authors:** Imran Rashid Rangraze, Mohamed El-Tanani, Adil Farooq Wali, Rasha Babiker, Syed Arman Rabbani, Ismail I Matalka, Shakta Mani Satyam, Ashot Avagimyan, Karolina Hoffmann, Ioannis Ilias, Sorina Ispas, Maggio Viviana, Anna Paczkowska, Manfredi Rizzo

**Affiliations:** 1 RAS AL KHAIMAH (RAK) College of Medical Sciences, RAK Medical and Health Sciences University, Ras Al Khaimah, United Arab Emirates; 2 RAS AL KHAIMAH (RAK) College of Pharmacy, RAK Medical and Health Sciences University, Ras Al Khaimah, United Arab Emirates; 3 RAS AL KHAIMAH (RAK) Medical and Health Sciences University, Ras Al Khaimah, United Arab Emirates; 4 Yerevan State Medical University after M. Heratsi, Yerevan, Armenia; 5 Isfahan Cardiovascular Research Centre, Cardiovascular Research Institute, Isfahan University of Medical Sciences, Isfahan, Iran; 6 Department and Clinic of Internal Diseases and Metabolic Disorders, Poznan University of Medical Sciences, Poznań, Poland; 7 Department of Endocrinology, Diabetes and Metabolism, Elena Venizelou Hospital, Athens, Greece; 8 Department of Anatomy, Faculty of General Medicine, “Ovidius” University, Constanta, Romania; 9 School of Medicine, PROMISE Department of Health Promotion Sciences Maternal and Infantile Care, Internal Medicine and Medicinal Specialties, University of Palermo, Palermo, Italy; 10 Department of Pharmacoeconomics and Social Pharmacy, Poznan University of Medical Sciences, Poznań, Poland

**Keywords:** AI diagnostics, biomarkers, exocrine pancreatic insufficiency, pancreatogenic diabetes, precision medicine, reclassification, type 5 diabetes mellitus

In the published article, there was a mistake in the title. The title of this article was erroneously given as “*Type 5 diabetes mellitus: redefining pancreatogenic diabetes through molecular, imaging, and AI-driven evidence*”. The correct title of the article is “*Redefining Pancreatogenic Diabetes Mellitus Through Molecular, Imaging, and AI-Driven Evidence*”.

In the published article, the **Abstract** mentioned type 5 diabetes mellitus. We have revised the **Abstract** to match with the rest of the article. The **Abstract** appeared as follows:

“Background: Type 5 Diabetes Mellitus (T5DM), denoting pancreatogenic diabetes from fibro-inflammatory pancreatic injury, is a distinct yet under-recognised entity. Current WHO and ADA classifications overlook its complex, concurrent endocrine–exocrine failures, contributing to misdiagnosis, treatment gaps, and suboptimal outcomes.

Objectives: This review aims to critically analyze current scientific understanding of the pathogenesis, diagnostic criteria, metabolic consequences, and therapeutic needs of T5DM and suggest a precise framework of medicine that justifies the need for T5DM to be formally recognized as a sub-type of diabetes.

Methods: An integrative review was conducted using recent literature on pancreatic pathophysiology, molecular biomarkers, radiomics, diagnostic imaging, glycemic control technologies, and machine learning. The focus was on the recent literature to elucidate the biological, diagnostic, and treatment aspects of the clinical studies, guidelines, and mechanistic research available from the publications.

Key findings: T5DM involves loss of insulin and glucagon alongside exocrine pancreatic insufficiency, malnutrition, and significant glycaemic variability. A tiered diagnostic framework—integrating pancreatic imaging, endocrine–exocrine testing, autoimmune exclusion, and emerging biomarkers—enhances accuracy. Management requires coordinated hormonal and enzyme replacement, structured nutritional support, and targeted surveillance for malignancy and micronutrient deficits. Radiomics, quantitative imaging, and AI-driven analytics offer valuable tools for earlier detection, improved risk stratification, and personalised therapy.

Conclusion: T5DM warrants recognition as a distinct diabetes entity owing to its unique pathophysiology, clinical behaviour, and therapeutic needs. Harmonised diagnostic criteria, validated biomarker and imaging pathways, and multicentre registries are essential to integrate T5DM into global classification systems and advance mechanism-based, personalised care.”

This has been corrected to read:

“Background: Pancreatogenic Diabetes Mellitus (PDM), denoting diabetes secondary to fibro- inflammatory and structural pancreatic injury, represents a distinct yet under-recognised clinical entity. Current classification systems frequently subsume this condition under ―other specific types‖ of diabetes, contributing to misdiagnosis, therapeutic misdirection, and suboptimal outcomes.

Objectives: This review critically analyses contemporary evidence regarding the pathogenesis, diagnostic framework, metabolic consequences, and therapeutic requirements of pancreatogenic diabetes. We propose a mechanism-based, precision-oriented clinical model integrating structural, functional, and emerging biomarker evidence.

Methods: An integrative review of recent literature was conducted, focusing on pancreatic pathophysiology, endocrine–exocrine interplay, diagnostic imaging modalities, molecular biomarkers, radiomics, and artificial intelligence–assisted analytics.

Key Findings: Pancreatogenic Diabetes Mellitus is characterised by progressive insulin deficiency, impaired glucagon counter-regulation, and exocrine pancreatic insufficiency, often accompanied by malnutrition and glycaemic instability. A structured diagnostic framework integrating pancreatic imaging, endocrine–exocrine functional testing, and autoimmune exclusion improves classification accuracy.

Management requires coordinated insulin therapy, pancreatic enzyme replacement, nutritional optimisation, and surveillance for skeletal and malignant complications. Emerging radiomic and AI- driven tools may enhance early detection and risk stratification, although prospective validation remains necessary.

Conclusion: Pancreatogenic Diabetes Mellitus warrants clearer mechanistic recognition within global diabetes classification frameworks. Harmonised diagnostic criteria, translational research integration, and multidisciplinary care pathways are essential to improve patient outcomes and advance precision medicine in this domain.

Type 5 Diabetes mellitus was used throughout the article. A correction has been made and type 5 diabetes mellitus has been replaced with Pancreatogenic diabetes mellitus (PDM) in all the sections, tables and figures.”

The original version of this article has been updated.

